# Association between cagA negative *Helicobacter pylori* status and nonalcoholic fatty liver disease among adults in the United States

**DOI:** 10.1371/journal.pone.0202325

**Published:** 2018-08-15

**Authors:** Seung Joo Kang, Hwa Jung Kim, Donghee Kim, Aijaz Ahmed

**Affiliations:** 1 Department of Internal Medicine, Healthcare Research Institute, Seoul National University, Hospital Healthcare System Gangnam Center, Seoul, Korea; 2 Department of Clinical epidemiology and Biostatistics, Asan Medical Center, University of Ulsan College of Medicine, Seoul, Korea; 3 Division of Gastroenterology and Hepatology, Stanford University School of Medicine, Stanford, CA, United States of America; Institute of Medical Research A Lanari-IDIM, University of Buenos Aires-National Council of Scientific and Technological Research (CONICET), ARGENTINA

## Abstract

We investigated the relationship of *H*. *pylori* stratified by cytotoxin-associated gene A (cagA) status with nonalcoholic fatty liver disease (NAFLD) in the general population of the United States (US). We utilized the Third National Health and Nutrition Examination Survey from 1988 to 1994 in this study. NAFLD was defined by ultrasonographic detection of hepatic steatosis in the absence of other known causes of liver diseases and significant alcohol consumption. Hepatic steatosis was assessed by parenchymal brightness, liver to kidney contrast, deep beam attenuation, bright vessel walls and gallbladder wall definition. Antibodies to *H*. *pylori* and cagA of participants were measured using *H*. *pylori* IgG and anti-cagA IgG enzyme-linked immunosorbent assays. Among 5,404 participants, the prevalence of NAFLD was higher in *H*. *pylori* positive subjects (33.5±1.8%) compared to *H*. *pylori* negative subjects (26.1±1.7%, *p* <0.001). In terms of cagA protein status stratification, while cagA positive *H*. *pylori* group did not demonstrate an association with NAFLD (OR: 1.05; 95% CI: 0.81–1.37), cagA negative *H*. *pylori* group was noted to have a significant association with NAFLD in a multivariable analysis (OR: 1.30; 95% CI: 1.01–1.67). In conclusion, our study demonstrated that cagA negative *H*. *pylori* infection was an independent predictor of NAFLD in the US general population.

## Introduction

Nonalcoholic fatty liver disease (NAFLD) is the most prevalent liver disease in the United States (US) and in many parts of the world [1,2]. NAFLD is a clinical and pathological entity that encompasses nonalcoholic fatty liver and nonalcoholic steatohepatitis complicated by progressive stages of fibrosis, cirrhosis, and hepatocellular carcinoma [3]. Because the mechanism underlying the development of NAFLD has been linked to insulin resistance and metabolic syndrome, NAFLD is associated with many risk factors of cardiovascular disease, such as obesity, diabetes, and dyslipidemia [4–7].

*Helicobacter pylori* (*H*. *pylori*) infection has been associated with a variety of extra-digestive conditions, including cardiovascular, lung, hematologic, neurologic and hepatobiliary diseases [8,9]. In the Finnish Alpha-Tocopherol, Beta-Carotene Cancer Prevention (ATBC) study, *H*. *pylori* seropositivity was associated with an increased risk of biliary tract cancer [10]. Bacterial infection of intestinal tract may compromise mucosal integrity, allowing passive or facilitated entry of harmful bacteria or their products into the circulation [11]. Notably, cytotoxin-associated gene A (cagA) positive strains can introduce *H*. *pylori* proteins into the epithelial cells and induce an antibody response to the cagA protein. These strains was found to be associated with an increased risk of gastric cancer and peptic ulcer disease and a decreased risk of esophageal reflux disease [12]. Until now, several small-scale epidemiological studies have reported a relationship between NAFLD and *H*. *pylori* infection [8,13]. However, association between NAFLD and *H*. *pylori* infection with regard to cagA status has not been studied in a large, general population.

Therefore, in this study, we used the National Health and Nutrition Examination Survey (NHANES) data to investigate the impact of *H*. *pylori* infection with regard to cagA status on NAFLD.

## Methods

### Study population and study design

**Fig 1:**
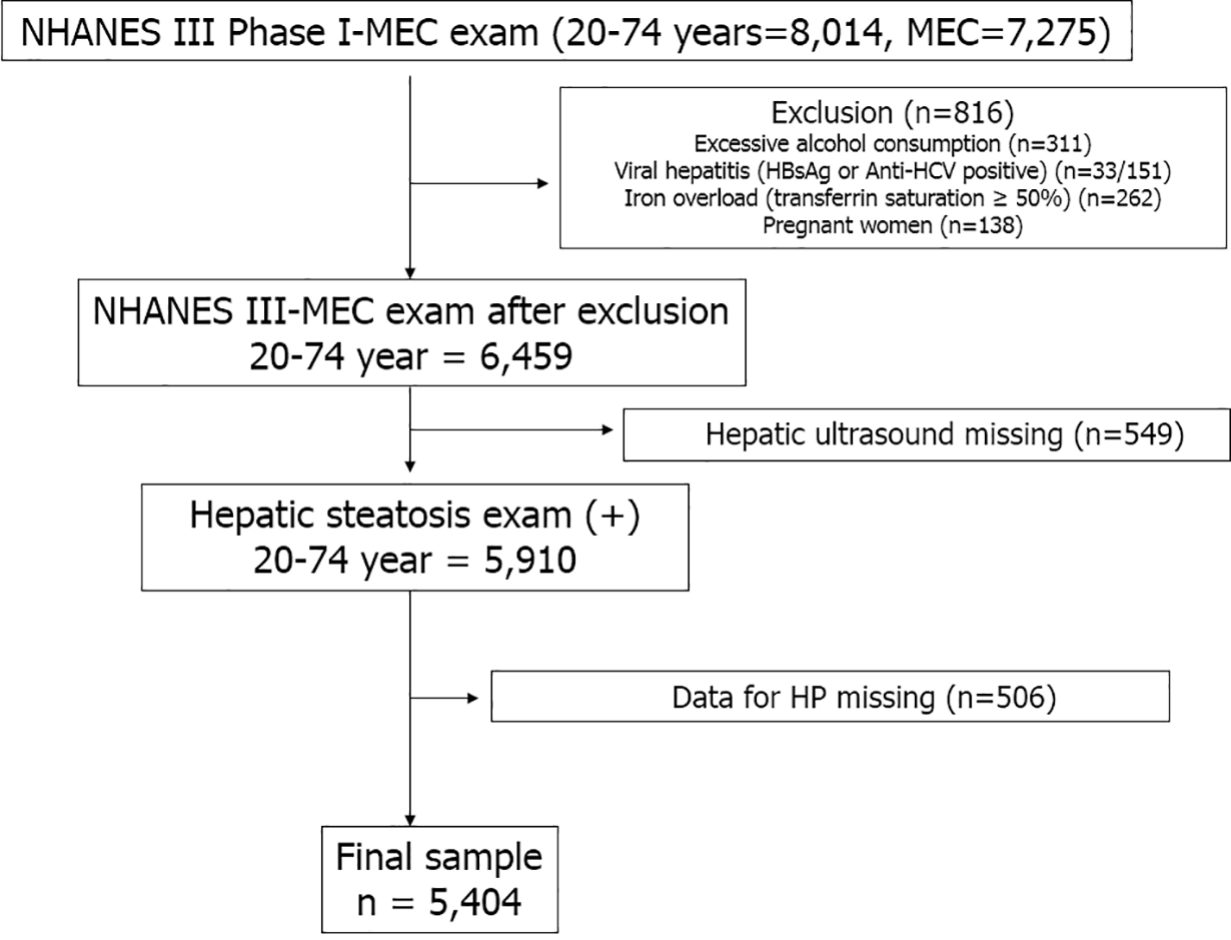
Flow diagram of participants for the study.

This study represents an analysis of the third NHANES data (1988–1994, the National Center for Health Statistics, the Centers for Disease Control and Prevention [CDC]). NHANES employed a stratified, multistage, clustered probability sampling design to obtain a representative sample of the non-institutionalized civilian population in the US. In total, 7,275 adults from 20 to 74 years of age, who participated in the NHANES III survey Phase I, underwent laboratory tests at a mobile examination center (Fig 1). Subjects with significant alcohol consumption (> 21 drinks/week in men and > 14 drinks/week in women) [14], viral hepatitis (positive serum hepatitis B surface antigen and/or positive serum hepatitis C antibody), iron overload (transferrin saturation ≥ 50%), and pregnant women were excluded (n = 816). Among the remaining 6,459 participants, hepatic steatosis could be evaluated in 5,910 (91.5%). We further excluded participants whose tests did not examine *H*. *pylori* status. Finally, 5,404 subjects were included and analyzed in this study. The original NHANES survey was approved by the CDC’s Institutional Review Board, and all participants provided written informed consent to participate. This retrospective study was exempted by the Institutional Review Board of the Seoul National University Hospital because the dataset used in this analysis was completely deidentified. This study was conducted according to the Guideline for Good Clinical Practice (GCP) and the provisions of the Helsinki declaration.

### *H*. *pylori* status

Antibodies to *H*. *pylori* were measured in participants 20 years and older using a *H*. *pylori* IgG enzyme-linked immunosorbent assay (ELISA) (Wampole Laboratories, Cranbury, New Jersey) in serum surplus samples [15]. Additionally, anti-cagA IgG was also measured in surplus sera among participants positive for *H*. *pylori*, using a method developed and standardized by Vanderbilt University, as described previously [16]. On the basis of the *H*. *pylori* and cagA status, we classified participants into three groups: *H*. *pylori* positive and cagA positive, *H*. *pylori* positive and cagA negative, and *H*. *pylori* negative [17].

### Assessment of NAFLD

We used a previously described method to assess NAFLD [2]. Briefly, the original NHANES III survey included gallbladder ultrasonography as part of the assessment for digestive disease in adults aged 20 and above. Three board-certified radiologists reviewed the archived gallbladder ultrasonography images to assess fatty liver [18]. Assessment of fatty liver was performed using the following criteria: (1) parenchymal brightness; (2) liver to kidney contrast; (3) deep beam attenuation; (4) bright vessel walls; and (5) gallbladder wall definition. Overall assessment, made using an algorithm based on these five criteria, defined normal versus mild, moderate, or severe hepatic steatosis [18]. For our study, NAFLD was diagnosed as the presence of any degree of fatty liver (mild to severe).

### Advanced fibrosis by noninvasive panels

The noninvasive panels used for the evaluation of advanced fibrosis have been previously described [2, 19]. Briefly, advanced fibrosis was assessed by NAFLD fibrosis score, FIB-4 score, and aspartate aminotransferase to platelet ratio index. Due to the small sample size of subjects with high probability of advanced fibrosis, ‘suspected advanced fibrosis’ was evaluated and defined as presence of high probability for advanced fibrosis on at least one of the three non-invasive fibrosis panels.

### Clinical and laboratory evaluation

A vast array of demographic, lifestyle, dietary, anthropometric and comprehensive laboratory data were available in this NHANES dataset. Hypertension was defined as systolic blood pressure ≥ 140 mmHg or diastolic blood pressure ≥ 90 mmHg and/or previous use of antihypertensive medication. Diabetes mellitus was defined as having a history of diabetes and/or treatment with a hypoglycemic agent or insulin. Current smokers were participants who reported ongoing smoking or those who had smoked at least 100 cigarettes in the preceding 5 years.

### Statistical analyses

The outcome variable in this study was the presence of NAFLD. Analyses were conducted using the SAS-callable SUDAAN 10.0.1 (Research Triangle Institute, Research Triangle Park, NC), which allows for the stratified sampling scheme by NHANES to project these data to the US population [20]. We analyzed proportions of categorical variables and means ± standard error of continuous variables (PROC CROSSTAB, PROC DESCRIPT). We used chi-squared tests for the categorical variables and Student’s t-test and one-way analysis of variance (ANOVA) for the continuous variables (PROC CROSSTAB, PROC REGRESS). A multivariable logistic regression model was used to investigate the independent association between NAFLD and *H*. *pylori* status (PROC LOGISTIC). All subjects in NHNAES III aged 17 years and over were followed for mortality through December 2006. We utilized Cox proportional hazards regression testing for survival analysis of overall mortality.

## Results

### Baseline characteristics according to NAFLD

The prevalence of NAFLD among the eligible subjects was 31.9%. Demographic and clinical characteristics of subjects with NAFLD are shown in Table 1. Subjects with NAFLD were more likely to be older, male, hypertensive, and diabetic than those without NAFLD. Similarly, body mass index (BMI), waist circumference, lipid panels, liver enzymes, and fasting glucose were higher in subjects with NAFLD than those without NAFLD.

**Table 1:** Baseline characteristics according to presence of NAFLD (NHANES 1988–1994, n = 5,404).

	NAFLD (n = 1,723)	No NAFLD (n = 3,681)	P value

Age (years)	46.1 ± 0.15	41.5 ± 0.18	<0.001
Gender (male, %)	49.7 ± 0.31	45.8 ± 0.38	<0.001
Body mass index (kg/m^2^)	29.2 ± 0.10	25.3 ± 0.05	<0.001
Waist circumference (cm)	99.1 ± 0.25	88.5 ± 0.15	<0.001
Race-ethnicity (%)			<0.001
Non-Hispanic white	77.2 ± 0.86	78.0 ± 0.56	
Non-Hispanic black	9.2 ± 0.20	10.4 ± 0.37	
Mexican American	6.5 ± 0.35	4.8 ± 0.15	
Other Race	7.2 ± 0.56	6.7 ± 0.47	
Smoking (%)			<0.001
Never	25.8 ± 0.38	31.5 ± 0.50	
Ex-smoker	29.8 ± 0.38	24.8 ± 0.33	
Current smoking	44.3 ± 0.61	43.7 ± 0.50	
Alcohol consumption (drinks/wk)	2.5 ± 0.05	2.7 ± 0.04	0.399
Hypertension (%)	34.5 ± 0.62	16.1 ± 0.36	<0.001
Diabetes (%)	9.3 ± 0.13	2.7 ± 0.08	<0.001
History of cardiovascular disease (%)	6.6 ± 0.23	3.1 ± 0.13	<0.001
Lipid-lowering medication (%)	4.7 ± 0.23	2.2 ± 0.13	<0.001
Systolic blood pressure (mm Hg)	125.6 ± 0.21	119.4 ± 0.17	<0.001
Diastolic blood pressure (mm Hg)	76.4 ± 0.14	73.0 ± 0.10	<0.001
Total cholesterol (mg/dL)	211.6 ± 0.23	202.3 ± 0.42	<0.001
HDL cholesterol (mg/dL)	47.8 ± 0.17	52.3 ± 0.17	<0.001
Triglycerides (mg/dL)	178.7 ± 1.18	121.7 ± 1.19	<0.001
ALT (IU/L)	19.1 ± 0.21	14.3 ± 0.07	<0.001
AST (IU/L)	22.5 ± 0.13	19.7 ± 0.07	<0.001
GGT (IU/L)	32.5 ± 0.47	23.4 ± 0.12	<0.001
Albumin (g/dL)	4.31 ± 0.01	4.30 ± 0.01	0.401
Platelet (x10^9^/L)	286.3 ± 0.53	280.6 ± 0.45	0.137
Glucose (mg/dL)	106.2 ± 0.42	95.2 ± 0.19	<0.001
Vitamin D	27.7 ± 0.11	30.1 ± 0.10	<0.001
HbA1c (%)	5.5 ± 0.01	5.2 ± 0.01	<0.001
HP positive (%)	38.2 ± 0.40	30.3 ± 0.52	<0.001
CagA positive (%)	19.2 ± 0.38	17.0 ± 0.31	<0.001
CagA negative (%)	19.0 ± 0.16	13.3 ± 0.33	

Abbreviation: NAFLD, nonalcoholic fatty liver disease; HDL, high-density lipoprotein; ALT, alanine aminotransferase; AST, aspartate aminotransferase; GGT, gamma-glutamyltransferase.

Data are expressed as the mean ± standard error or proportion ± standard error

### Baseline characteristics according to *H*. *pylori* status

Table 2 shows the distribution of participants based on *H*. *pylori* status by demographic and clinical risk factors in the overall study population, including those with data on *H*. *pylori* cagA status. Subjects with positive *H*. *pylori* serology results were more likely to be older, male and with a higher BMI and waist circumference than that of *H*. *pylori*-negative participants. Subjects of a race/ethnicity other than non-Hispanic white and those who had a history of diabetes or hypertension were more likely to be *H*. *pylori*-positive. Similarly, lipid panels, liver enzymes, and fasting glucose were higher in subjects with positive *H*. *pylori* serology than without. There was no apparent association of *H*. *pylori* positivity with current smoking status. Among subjects with positive *H*. *pylori* serology, those without cagA were likely to be older with higher prevalence of hypertensive versus those with cagA. Subjects of a race/ethnicity other than non-Hispanic white were more likely to be cagA positive.

**Table 2:** Baseline characteristics of study participants based on *H*. *pylori* status (NHANES 1988–1994, n = 5,404).

	*H*. *pylori-* (*n* = 2,749)	*H*. *pylori+* (*n* = 2,655)	*H*. *pylori+*	
			CagA+ (*n* = 1,617)	CagA- (*n* = 1,038)
Age (years)	40.2 ± 0.12	48.4 ± 0.26*	47.1 ± 0.34	49.9 ± 0.24^†^
Gender (male, %)	46.1 ± 0.36	48.5 ± 0.52*	49.7 ± 0.98	47.0 ± 0.82
Body mass index (kg/m^2^)	26.0 ± 0.07	27.2 ± 0.06*	27.1 ± 0.07	27.3 ± 0.10
Waist circumference (cm)	90.3 ± 0.19	94.1 ± 0.20*	93.4 ± 0.25	94.8 ± 0.26
Race-ethnicity (%)				
Non-Hispanic white	85.1 ± 0.48	62.7 ± 0.87*	51.3 ± 1.57	76.2 ± 0.43^††^
Non-Hispanic black	7.0 ± 0.28	16.4 ± 0.36	23.0 ± 0.39	8.6 ± 0.29
Mexican American	2.9 ± 0.11	10.1 ± 0.40	11.3 ± 0.64	8.6 ± 0.38
Other Race	4.9 ± 0.38	10.8 ± 0.62	14.4 ± 1.15	6.6 ± 0.25
Smoking (%)				
Never	30.0 ± 0.42	29.6 ± 0.72	29.7 ± 1.00	29.5 ± 0.66
Ex-smoker	25.9 ± 0.41	26.9 ± 0.33	26.6 ± 0.46	27.2 ± 0.43
Current smoking	44.1 ± 0.67	43.5 ± 0.64	43.7 ± 0.98	43.3 ± 0.76
Alcohol consumption	2.9 ± 0.05	2.0 ± 0.05*	1.9 ± 0.08	2.1 ± 0.06
Hypertension (%)	17.2 ± 0.38	29.9 ± 0.50*	27.6 ± 0.56	32.6 ± 0.64^††^
Diabetes (%)	3.8 ± 0.08	6.2 ± 0.16*	6.2 ± 0.29	6.2 ± 0.27
History of cardiovascular disease (%)	2.9 ± 0.15	6.5 ± 0.18*	6.8 ± 0.33	6.1 ± 0.12
Lipid-lowering medication (%)	2.6 ± 0.14	3.5 ± 0.12*	3.1 ± 0.14	4.0 ± 0.21^††^
Systolic blood pressure (mm Hg)	119.2 ± 0.16	125.5 ± 0.20*	124.7 ± 0.31	126.4 ± 0.24
Diastolic blood pressure (mm Hg)	73.2 ± 0.13	75.6 ± 0.11*	75.8 ± 0.18	75.4 ± 0.10
Total cholesterol (mg/dL)	201.8 ± 0.42	211.4 ± 0.57*	210.4 ± 0.67	212.5 ± 0.71
HDL cholesterol (mg/dL)	51.6 ± 0.17	49.8 ± 0.24*	49.7 ± 0.31	49.9 ± 0.24
Triglycerides (mg/dL)	133.6 ± 1.15	147.2 ± 1.42*	142.4 ± 2.21	153.0 ± 1.39
ALT (IU/L)	15.5 ± 0.12	16.1 ± 0.16	16.0 ± 0.24	16.2 ± 0.16
AST (IU/L)	20.0 ± 0.05	21.4 ± 0.12*	21.4 ± 0.19	21.4 ± 0.11
GGT (IU/L)	24.6 ± 0.14	28.3 ± 0.44*	29.3 ± 0.87	27.1 ± 0.25^††^
Albumin (g/dL)	4.33 ± 0.00	4.26 ± 0.01*	4.2 ± 0.01	4.3 ± 0.01
Platelet (x10^9^/L)	282.9 ± 0.48	280.9 ± 0.72	280.5 ± 1.25	281.3 ± 0.65
Glucose (mg/dL)	96.5 ± 0.27	102.2 ± 0.20*	102.9 ± 0.40	101.4 ± 0.21
Vitamin D	30.3 ± 0.07	27.6 ± 0.18*	27.1 ± 0.18	28.1 ± 0.25
HbA1c (%)	5.2 ± 0.01	5.5 ± 0.01*	5.5 ± 0.01	5.5 ± 0.01

Abbreviation: *H*. *pylori*, *Helicobacter pylori*; NAFLD, nonalcoholic fatty liver disease; HDL, high-density lipoprotein; ALT, alanine aminotransferase; AST, aspartate aminotransferase; GGT, gamma-glutamyltransferase. Data are expressed as the mean ± standard error or proportion ± standard error

* *p* <0.01 *H*. *pylori+ vs*. *H*. *pylori-*

^†^ *p* <0.05 *H*. *pylori+* cagA*+ vs*. *H*. *pylori-* cagA-

^††^ *p* <0.01 *H*. *pylori+* cagA*+ vs*. *H*. *pylori-* cagA-

### NAFLD prevalence according to *H*. *pylori* status

**Fig 2:**
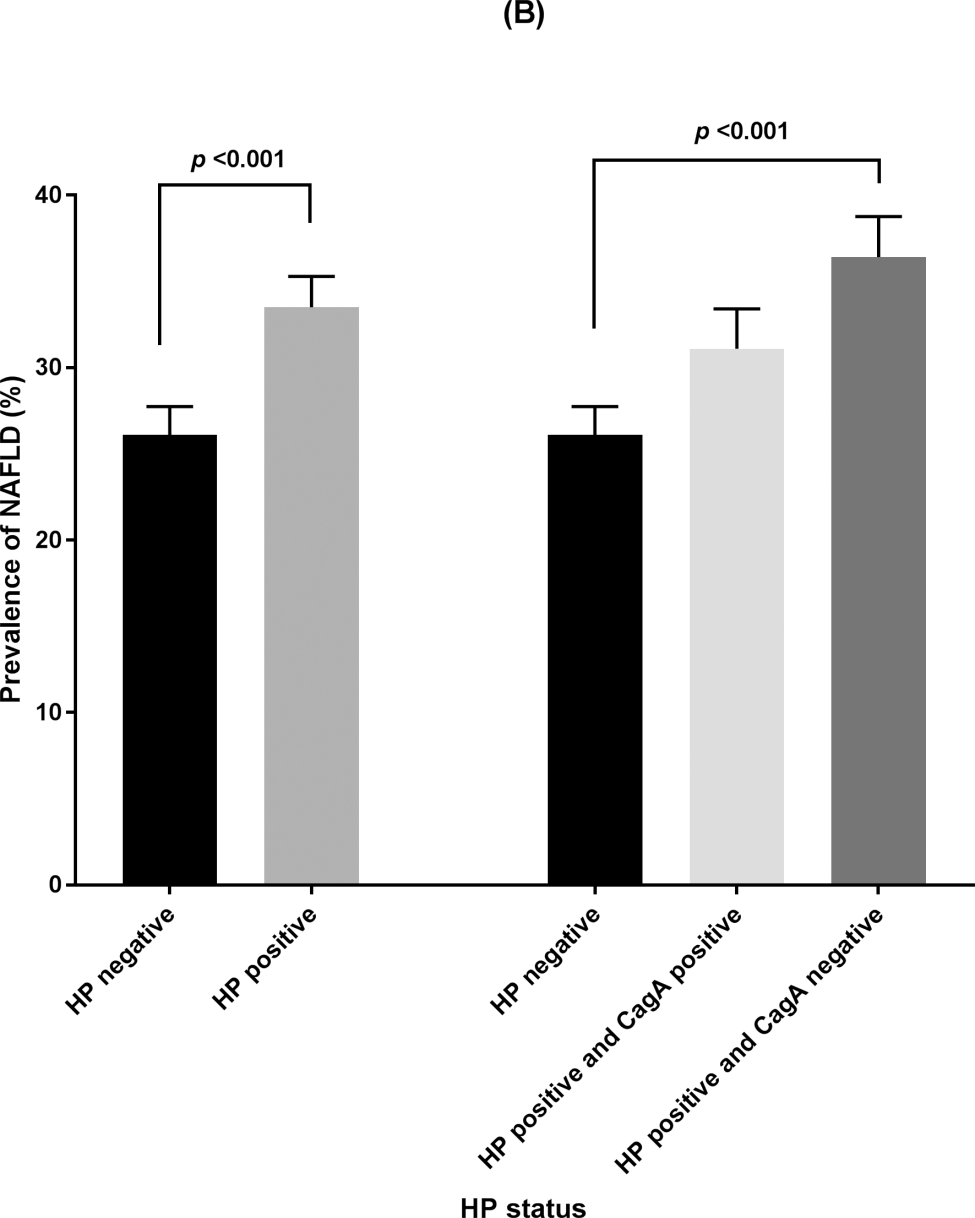
Prevalence of NAFLD defined by ultrasonography according to *H*. *pylori* and cagA serologic tests. HP, *H*. *pylori*; NAFLD, nonalcoholic fatty liver disease.

The prevalence of NAFLD was significantly higher in the *H*. *pylori* positive subjects than that in the *H*. *pylori* negative subjects (33.5 ± 1.8% vs. 26.1 ± 1.7%, *p* <0.001). Among *H*. *pylori* positive subjects, the prevalence of NAFLD was significantly higher in the cagA negative subjects than in the cagA positive subjects (36.4 ± 2.4% vs. 31.1 ± 2.3%, *p* <0.001) (Fig 2). Subjects with NAFLD showed positive relationships with *H*. *pylori* positivity in the univariate analysis (OR: 1.43, 95% CI: 1.23–1.66). However, after adjustment for known metabolic risk factors, multivariate regression analysis showed that this association was attenuated (OR: 1.17, 95% CI: 0.95–1.43) with marginal significance. To identify the influence of cagA on NAFLD, we performed an analysis based on cagA status. NAFLD was significantly associated with the cagA positive group (OR: 1.28, 95% CI: 1.07–1.53) and cagA negative group (OR: 1.62, 95% CI: 1.30–2.03) in univariate analysis. CagA positivity was not significantly associated with NAFLD after adjustment of multiple risk factors (OR: 1.05, 95% CI: 0.81–1.37). However, the cagA negative *H*. *pylori* positive group was significantly associated with NAFLD in multivariable regression analysis (OR: 1.30, 95% CI: 1.01–1.67) after adjustment (Table 3). The effect size of cagA negative *H*. *pylori* positivity was similar to that of Mexican Americans (OR 1.29; 95% CI 1.02–1.63), however, much lower than that of diabetes (OR 2.03; 95% CI 1.37–3.01) and hypertension (OR 1.67; 95% CI 1.26–2.19) (S1 Table).

**Table 3:** Univariate and multivariable analyses of the risk for NAFLD according to *H*. *pylori* status.

	Univariate		Multivariable-adjusted	
	OR (95% CI)	*P* Value	OR (95% CI)	*P* Value
HP positivity				
Negative	Reference		Reference	
Positive	1.43 (1.23–1.66)	<0.001	1.17 (0.95–1.43)	0.139
HP and Cag A positivity				
Negative	Reference		Reference	
CagA positive	1.28 (1.07–1.53)	0.008	1.05 (0.81–1.37)	0.715
CagA negative	1.62 (1.30–2.03)	<0.001	1.30 (1.01–1.67)	0.044

Abbreviation: NAFLD, nonalcoholic fatty liver disease; *H*. *pylori*, *Helicobacter pylori*; OR, odds ratio; CI, confidence interval.

Multivariable models adjusted for age, sex, race-ethnicity, income, diabetes, hypertension, smoking status, waist circumference, alcohol consumption, caffeine consumption, total cholesterol, high-density lipoprotein-cholesterol, and transferrin saturation.

When we evaluated the association of *H*. *pylori* seropositivity and/or cagA presence with advanced fibrosis among subjects with NAFLD (S2 Table), no statistically significant associations were noted in the univariate and multivariable models. As shown in S3 Table, *H*. *pylori* positivity was associated with significantly higher hazards of all-cause mortality, and the adjusted hazard ratio (HR) and 95% confidence interval (CI) was 1.95 (1.49–2.55) for univariate model and 1.30 (1.01–1.68) for multivariate model 1. With further adjustment for traditional risk factors, this association was attenuated and demonstrated only marginal significance (P for trend = 0.138). The cagA negative or positive *H*. *pylori* positive group with NAFLD demonstrated significant association with increased risk of all-cause mortality in univariate model. However, this association remained insignificant in multivariate model.

**Fig 3:**
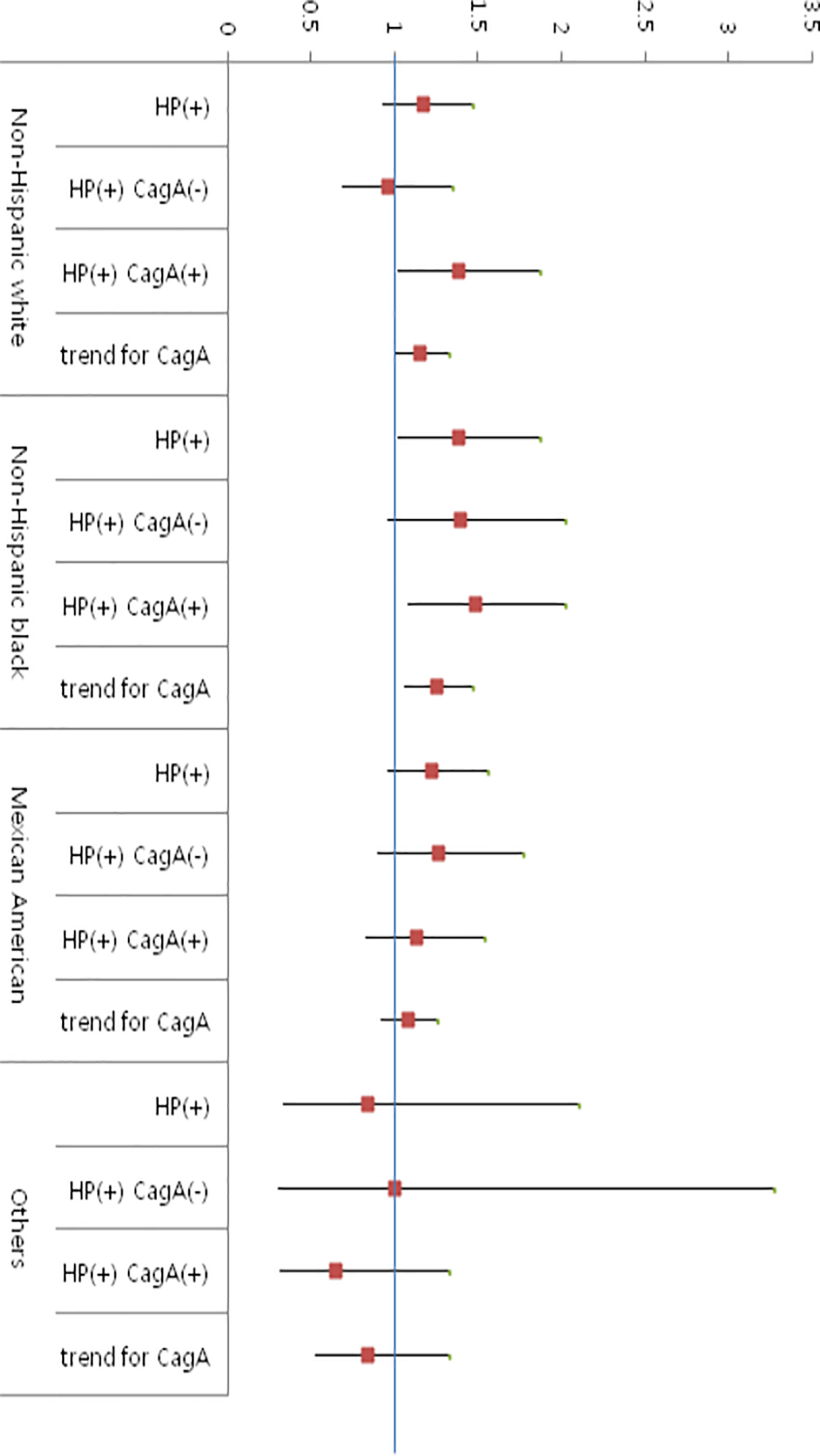
Association between NAFLD and *H*. *pylori* status according to race-ethnicity.

*H*. *pylori* infection was associated with an increased risk of NAFLD in all ethnicities. However, *H*. *pylori* infection was significantly associated with NAFLD in non-Hispanic black participants. Regarding cagA status, cagA negative *H*. *pylori* was associated with an increased risk for NAFLD in the non-Hispanic white and non-Hispanic black populations. However, cagA negative *H*. *pylori* status was not associated with NAFLD in Mexican-Americans (Fig 3).

## Discussion

The main finding in this nationally representative, population-based study was that the prevalence of NAFLD was higher in subjects with *H*. *pylori* than in those without. In subjects with *H*. *pylori*, the prevalence of NAFLD was greater in cagA negative subjects compared to cagA positive subjects. Additionally, cagA negative *H*. *pylori* positivity was significantly associated with NAFLD after adjustment for multiple classical risk factors, confirming that a relevant clinical relationship exists between these two conditions.

Several studies have investigated the association between *H*. *pylori* and NAFLD. Polyzos et al. reported that higher rates of anti-*H*. *pylori* IgG were observed in the NAFLD group (n = 28) compared to that of the healthy control group (n = 25) [8]. They also showed that biopsy-proven NAFLD patients had higher insulin and tumor necrosis factor (TNF)-α levels compared to those of controls. However, there were no significant differences in steatosis grade, fibrosis stage, lobular or portal inflammation, or balloon degeneration when NAFLD patients were divided based on *H*. *pylori* IgG seropositivity or ^13^C urea breath test positivity [8]. This study was also limited by a small sample size. Dogan et al. showed that fatty liver determined by ultrasonographic score was observed more frequently in *H*. *pylori* positive patients, but they did not consider other metabolic confounders [13]. NAFLD is considered as the hepatic manifestation of metabolic syndrome and is strongly associated with cardiovascular disease. [21]. Several studies reported that an association between *H*. *pylori* and metabolic syndrome is significant as shown in case of NAFLD. In a large cross-sectional study from Japan, *H*. *pylori* seropositivity was significantly associated with metabolic syndrome [22]. On the contrary, studies from highly endemic areas of *H*. *pylori* revealed no association between positive *H*. *pylori* serology and NAFLD [23,24]. In a recent meta-analysis, *H*. *pylori* infection and NAFLD demonstrated a modest, significant association (pooled OR 1.21 (95% CI 1.07–1.37) [25]. Recently, Fan et al. found that prevalence of NAFLD was significantly higher in subjects with *H*. *pylori* infection (36.0% vs. 33.3%, *p* <0.05) [26]. However, after adjusting for confounding factors, *H*. *pylori* infection was not significantly associated with NAFLD. In our study using representative US population, we found *H*. *pylori* and cagA negativity was associated with NAFLD, which may partly explain the inconsistent results in the literature.

In the present study, we found only infection with cagA negative H. pylori was significantly associated with NAFLD. CagA plays an important role not only in gastric inflammation but also in the development of gastric cancer. Regarding the effect of cagA on cardiovascular and metabolic risk factors, a German population-based cohort study showed decreased incidence of cardiovascular mortality in the cagA positive population compared with that of *H*. *pylori*-negative subjects. The difference of incidence between the two groups was 33% - 38%, but the cardiovascular mortality of the cagA negative population was similar to that of the *H*. *pylori*-negative group [27]. Analysis of the association between *H*. *pylori* and mortality in the NHANES III study showed an inverse relationship between mortality from stroke and cagA positive *H*. *pylori* infection [28]. The inverse relation between cagA and cardiovascular mortality can be explained by regulatory T cells (T-reg). In mice model, cagA plays an important role in the migration of CD4+ T cells in the gastric mucosa and cagA-dependent T-cell priming induces T-reg differentiation [29]. The presence of T-reg in the gastric mucosa of *H*. *pylori*-infected subjects suggested their involvement in suppressing mucosal immune responses, contributing to the infection persistence and modulation of the *H*. *pylori*-induced gastritis [30]. Reduced risk of asthma and allergies in *H*. *pylori*-infected populations appears to be related to the induction of T-reg responses [31]. T-reg is also known to be important in regulating inflammatory processes in NAFLD [31]. The highly stimulated regulatory immune system in the setting of cagA may be the reason why only cagA negative *H*. *pylori* infection is related with NAFLD as shown in this study.

Insulin resistance can be another possible explanation for the association between *H*. *pylori* and NAFLD. Insulin resistance is a key factor in both metabolic syndrome and NAFLD. One study showed that insulin resistance, which is measured as HOMA-IR, was significantly higher in the *H*. *pylori* positive group than in the *H*. *pylori* negative counterparts [32]. A systematic review of nine studies investigating the association between *H*. *pylori* infection and insulin resistance indicated that *H*. *pylori* infection was positively associated with insulin resistance [33]. It is well known that even chronic subclinical inflammation is associated with cardiovascular disease [34]. Long-term *H*. *pylori* infection induces a ‘state of inflammation’ by stimulating excessive release of pro-inflammatory cytokine and vasoactive substances, such as interleukin (IL)-6, IL-8, IL-1β, and TNF-α [35,36]. TNF-α is a key mediator of both direct and indirect effects of *H*. *pylori* infection on NAFLD. TNF-α interferes with insulin signaling, thereby favoring steatosis, and may play a pro-inflammatory role in the pathogenesis of NAFLD [37,38]. In addition, TNF-α can accelerate lipolysis, leading to an increase in free fatty acids. This can result in hepatocyte dysfunction, including increased oxidative stress, induction of endoplasmic reticulum dysregulation and subsequent expression of pro-inflammatory cytokines [39,40]. We noted that *H*. *pylori* positivity was associated with increased risk for all-cause mortality among subjects with NAFLD, though after adjusting for traditional risk factors, the association remained at marginal significance and thus needs further confirmation. This result perhaps suggests that the effect of *H*. *pylori* positivity in NAFLD on all-cause mortality may be mediated in part by diabetes, hypertension, and smoking.

In case of a disruption of the intestinal barrier, lipopolysaccharide from gram-negative bacteria increases in the portal circulation and is accompanied by increased levels of endotoxin-mediated cytokines in the liver. So bacterial constituents and cytokines enhance hepatic inflammation and fibrosis [41]. Miele et al. also found that in NAFLD patients, increased gut permeability and the prevalence of small intestine bacterial overgrowth correlated with the severity of steatosis but not with the presence of steatohepatitis [42]. *H*. *pylori* is believed to colonize not only the stomach and duodenal epithelium but also the biliary epithelium [43]. So *H*. *pylori* colonization in stomach and duodenum can increase in lipopolysaccharides and endotoxin in portal circulation and can promote hepatic inflammation and fibrosis.

The prevalence of *H*. *pylori* infection showed racial disparities. Both non-Hispanic blacks and Mexican-Americans had a significantly higher prevalence of *H*. *pylori* than non-Hispanic whites [44]. The prevalence of cagA positive *H*. *pylori* was higher in non-Hispanic blacks and Mexican-Americans versus non-Hispanic whites [44]. However, in this study, a significant association between cagA negative *H*. *pylori* strains and NAFLD was found in non-Hispanic whites and non-Hispanic blacks but not in Mexican-Americans. Further research is needed to confirm these ethnic differences.

Currently, only two studies have reported the effect of *H*. *pylori* eradication on metabolic profile or NAFLD. One prospective study revealed that in the group with successful eradication, levels of total adiponectin and each multimer form were significantly increased 12 weeks after completion of treatment [45]. This study suggested that eradication could have a beneficial effect on metabolic risk by increasing adiponectin levels. A recent small-scale randomized open label study showed that lifestyle modification plus *H*. *pylori* eradication did not result in significant differences in liver fat content by ultrasonography and in insulin resistance compared with that of lifestyle modification alone [46]. However, the primary outcome of this study is relatively subjective, and the open label design may be a pitfall. Therefore, further large prospective studies are needed to confirm the effect of *H*. *pylori* eradication on NAFLD.

There are strengths and limitations in our study. We studied a large representative sample from the US general population; thus, the results can be generalizable. Second, this study performed analysis according to cagA seropositivity and studied the differential impact of cagA presence (versus cagA absence) on the association between NAFLD and *H*. *pylori*. However, there may be other predictive factors that may play an influential role in impacting the association of *H*. *pylori* and NAFLD. Third, we included sufficient confounders from the NHANES III data, which increased the accuracy of this study. Finally, our study included various ethnic/racial groups, and thus, our conclusions may be applicable to the general population. This study also has some limitations. First, because of its cross-sectional design, a causal relationship between *H*. *pylori* infection and NAFLD could not be identified. Second, our diagnosis of NAFLD was based on ultrasonographic examination, which is not able to differentiate nonalcoholic steatohepatitis from nonalcoholic fatty liver. In addition, hepatic ultrasonography cannot identify fatty infiltration below 30% and has intra- and inter-observer variability in making a diagnosis. Third, our study is unable to offer a mechanistic explanation to associate cagA negative *H*. *pylori* seropositivity with the risk of NAFLD. Further studies are warranted to determine mechanism(s) explaining this association.

In conclusion, the present study suggests that *H*. *pylori* infection is related to an increased prevalence of NAFLD in the general population. CagA negative *H*. *pylori* infection was associated with an increased risk for NAFLD, confirming that a relevant clinical relationship exists between these two conditions. However, since pathogenic mechanisms have not been fully elucidated, future studies are needed to clearly understand the clinical implications of our results.

## Supporting information

S1 Table Univariate and multivariable analyses of the risk for advanced fibrosis among subjects with NAFLD according to *H*. *pylori* status. (DOCX)

S2 Table Univariate and multivariable cox-regression analyses of the risk for all-cause mortality among subjects with NAFLD according to *H*. *pylori* status. (DOCX)

S3 Table Multivariable analysis of the risk for NAFLD. (DOCX)
