# Supplementary material for: Association between cagA negative Helicobacter pylori status and nonalcoholic fatty liver disease among adults in the United States
Source: PLoS One. 2018 Aug 15;13(8):e0202325. doi: 10.1371/journal.pone.0202325 (PMC6093702; doi:10.1371/journal.pone.0202325)
Supplement: S1 Table — (DOCX) [file pone.0202325.s001.docx]

**Supplementary Table 1.** Multivariable Analysis of the Risk for NAFLD

|  | OR | P-value |
| --- | --- | --- |
| HP and CagA positivity |  |  |
| Negative | 1 |  |
| CagA positive | 1.05 (0.81-1.37) | 0.715 |
| CagA negative | 1.30 (1.01-1.67) | 0.044 |
| Age | 1.00 (0.99-1.01) | 0.708 |
| Sex | 1.22 (0.92-1.60) | 0.165 |
| Race/Ethnicity |  |  |
| Non-Hispanic white | 1 |  |
| Non-Hispanic black | 0.71 (0.48-1.04) | 0.077 |
| Mexican American | 1.29 (1.02-1.63) | 0.030 |
| Other Race | 1.33 (0.87-2.04) | 0.186 |
| Income | 0.93 (0.80-1.08) | 0.327 |
| Diabetes | 2.03 (1.37-3.01) | <0.001 |
| Hypertension | 1.67 (1.26-2.19) | <0.001 |
| Alcohol consumption | 1.01 (0.99-1.03) | 0.247 |
| Smoking | 1.07 (0.96-1.20) | 0.228 |
| Waist circumference | 1.05 (1.04-1.06) | <0.001 |
| Daily caffeine consumption | 1.00 (1.00-1.00) | 0.013 |
| Total cholesterol | 1.00 (1.00-1.00) | 0.807 |
| HDL cholesterol | 0.99 (0.99-1.00) | 0.129 |
| Transferrin saturation | 1.00 (0.99-1.01) | 0.675 |

Multivariable models adjusted for age, sex, race-ethnicity, income, diabetes, hypertension, smoking

status, waist circumference, alcohol consumption, caffeine consumption, total cholesterol, high-density lipoprotein-cholesterol, and transferrin saturation.
