# Supplementary material for: Association between cagA negative Helicobacter pylori status and nonalcoholic fatty liver disease among adults in the United States
Source: PLoS One. 2018 Aug 15;13(8):e0202325. doi: 10.1371/journal.pone.0202325 (PMC6093702; doi:10.1371/journal.pone.0202325)
Supplement: S2 Table — (DOCX) [file pone.0202325.s002.docx]

**Supplementary Table 2.** Univariate and Multivariable Analyses of the Risk for Advanced Fibrosis among Subjects with NAFLD according to *H. pylori* Status

|  | Univariate | | Multivariable-adjusted* | |
| --- | --- | --- | --- | --- |
|  | OR (95% CI) | *P* Value | OR (95% CI) | *P* Value |
| HP positivity |  |  |  |  |
| Negative | Reference |  | Reference |  |
| Positive | 1.40 (0.73-2.67) | 0.294 | 0.80 (0.42-1.53) | 0.489 |
| HP and CagA positivity |  |  |  |  |
| Negative | Reference |  | Reference |  |
| CagA Positive | 1.52 (0.62-2.65) | 0.283 | 0.85 (0.43-1.71) | 0.643 |
| CagA Negative | 1.28 (0.69-3.33) | 0.489 | 0.75 (0.34-1.69) | 0.474 |

Abbreviation: NAFLD, nonalcoholic fatty liver disease; *H. pylori,* *Helicobacter pylori*; OR, odds ratio; CI, confidence interval.

*Multivariable models adjusted for age, sex, race-ethnicity, diabetes, hypertension, smoking status, waist circumference, alcohol consumption, and caffeine consumption.
