# Supplementary material for: Association between cagA negative Helicobacter pylori status and nonalcoholic fatty liver disease among adults in the United States
Source: PLoS One. 2018 Aug 15;13(8):e0202325. doi: 10.1371/journal.pone.0202325 (PMC6093702; doi:10.1371/journal.pone.0202325)
Supplement: S3 Table — (DOCX) [file pone.0202325.s003.docx]

**Supplementary Table 3.** Univariate and Multivariable Cox-regression Analyses of the Risk for All-cause Mortality among Subjects with NAFLD according to *H. pylori* Status

|  | Univariate model | | Multivariable model 1 | | Multivariable model 2 | |
| --- | --- | --- | --- | --- | --- | --- |
|  | HR (95% CI) | *P* Value | HR (95% CI) | *P* Value | HR (95% CI) | *P* Value |
| HP positivity |  |  |  |  |  |  |
| Negative | Reference |  | Reference |  | Reference |  |
| Positive | 1.95 (1.49-2.55) | <0.001 | 1.30 (1.01-1.68) | 0.042 | 1.22 (0.93-1.60) | 0.138 |
| HP and CagA Positivity |  |  |  |  |  |  |
| Negative | Reference |  |  |  | Reference |  |
| CagA positive | 1.75 (1.18-2.62) | 0.008 | 1.31 (0.97-1.74) | 0.073 | 1.26 (0.93-1.71) | 0.133 |
| CagA negative | 2.15 (1.73-2.62) | <0.001 | 1.30 (0.97-1.74) | 0.077 | 1.20 (0.88-1.63) | 0.248 |

Abbreviation: NAFLD, nonalcoholic fatty liver disease; *H. pylori,* *Helicobacter pylori*; HR, hazard ratio; CI, confidence interval.

Multivariable models 1 was adjusted for age, sex, race-ethnicity, and waist circumference.

Multivariable model 2 was adjusted for age, sex, race-ethnicity, waist circumference, diabetes, hypertension, smoking status, alcohol consumption, and caffeine consumption.
